# Microfungus *Podosphaera fusca* and the Fungus-like Organism *Peronospora ficariae* as Potential Inhalant Allergens in a Mouse Model of Asthma

**DOI:** 10.3390/cells14120914

**Published:** 2025-06-17

**Authors:** Piotr Wlaź, Katarzyna Socała, Marta Palusińska-Szysz, Urszula Świderska, Dominika Szczypior, Magdalena Krasowska, Agnieszka Szuster-Ciesielska

**Affiliations:** 1 Department of Animal Physiology and Pharmacology, Institute of Biological Sciences, Maria Curie-Skłodowska University, Akademicka 19, 20-032 Lublin, Poland; piotr.wlaz@mail.umcs.pl (P.W.); katarzyna.socala@mail.umcs.pl (K.S.); 2 Department of Genetics and Microbiology, Institute of Biological Sciences, Maria Curie-Skłodowska University, Akademicka 19, 20-032 Lublin, Poland; marta.palusinska-szysz@mail.umcs.pl; 3 Department of Botany, Mycology and Ecology, Institute of Biological Sciences, Maria Curie-Skłodowska University, Akademicka 19, 20-032 Lublin, Poland; urszula.swiderska@mail.umcs.pl; 4 Department of Virology and Immunology, Institute of Biological Sciences, Maria Curie-Skłodowska University, Akademicka 19, 20-032 Lublin, Poland

**Keywords:** phytopathogenic microfungus, fungus-like organism, acute and chronic asthma, inflammatory response, mouse model

## Abstract

Allergic conditions have surged to unprecedented levels globally, affecting approximately 30% of the population. Fungi are among the major sources of allergens, accounting for about 6% of respiratory issues. Identifying the causes of respiratory allergies is not always possible. Our study assessed the capacity of two plant parasites, *Podosphaera fusca* and *Peronospora ficariae*, which infect *Cucurbita pepo* and *Ficaria verna*, to provoke inflammatory and asthmatic reactions in mouse models of acute and chronic asthma. We performed experiments by sensitizing mice through intranasal challenges with extracts from *P. fusca* and *P. ficariae*. Subsequently, we used ELISA tests to measure pro-inflammatory cytokines, including IL-4, IL-5, IL-13, TNF-α, and TGF-β. We evaluated specific IgE production through ELISA and examined histological changes in mouse lungs using hematoxylin-eosin staining. Our research revealed that *P. fusca* and *P. ficariae* induced significant production of all tested cytokines, increased specific IgE levels, and caused histological changes characteristic of acute and chronic asthma progression. Although weaker than the reference allergen ovalbumin, *P. fusca* and *P. ficariae* possess proinflammatory and asthma-inducing capabilities, indicating the potential to expand the current list of fungal allergens.

## 1. Introduction

Asthma, a significant noncommunicable disease (NCD), affects both children and adults. While children have higher rates of asthma incidence and prevalence, adults experience more severe morbidity and mortality [1]. According to the Australian Institute of Health and Welfare, asthma accounted for 35% of the total disease burden among all respiratory conditions in 2023 [2]. In 2022, the American Lung Association reported that 44.2 million Americans, or 13.5% of the population, had been diagnosed with asthma by a healthcare provider at some point, representing a 48% increase from 9.1% in 1999 [3]. In the European Union (EU), asthma diagnoses in 2015 were noted in 8.2% of adults and 9.4% of children [4].

Asthma is a long-term inflammatory condition marked by fluctuating airway constriction, which manifests clinically as repeated bouts of coughing, wheezing, and a feeling of tightness in the chest [5,6,7]. A key aspect of asthma is the airway blockage caused by a decrease in the diameter of the bronchi. This involves ongoing airway inflammation characterized by infiltrating and activating immune cells, such as dendritic cells, eosinophils, neutrophils, various lymphocyte subsets, innate lymphoid cells, and mast cells. The complex interactions between these cells and the airway lining result in the development of asthma symptoms [8,9,10]. A hallmark of asthma is the heightened sensitivity of the airways to various infectious and environmental stimuli.

Fungal spores have been recognized as significant inhalant allergens since the 12th century [11]. Although the precise prevalence of fungal allergies is unclear, it is estimated to affect from 3% to 10% of the population, with variations depending on local climate conditions [12]. Common fungi from the *Cladosporium*, *Penicillium*, *Aspergillus*, and *Alternaria* genera contain the best-documented allergenic properties [13]. *C. herbarum* and *A. alternata* rank as the third most common inhalant allergens, following house dust and grass pollen, underscoring the importance of evaluating the risks associated with airborne fungal spores [14]. These spores can cause IgE-mediated type I hypersensitivity reactions, such as allergic rhinitis and asthma. High levels of spores in the air are linked to increased hospitalizations and deaths related to asthma and a greater risk of developing rhinitis [12,15]. Other respiratory issues caused by fungi include bronchopulmonary mycoses, allergic sinusitis, and hypersensitivity pneumonitis [16,17]. However, standard skin or blood tests often fail to pinpoint the source of an existing allergy (allergen), suggesting that there may be unidentified allergens yet to be discovered.

In our previous experiments, utilizing widely accepted models of the upper (BEAS-2B cell line) and lower (A549 cell line) respiratory tracts [18,19,20], we were the first to demonstrate that the microfungus *Podosphaera fusca* (Fungi, Ascomycota), which infects *Cucurbita pepo*, and the fungus-like organism *Peronospora ficariae* (Chromista, Oomycota), a parasite of *Ficaria verna*, exhibit proinflammatory and proallergenic characteristics (data not yet published) confirmed by increased levels of proinflammatory cytokines (IL-4, IL-5, IL-13, TNF-α, and TGF-β) and decreased expression of tight junction proteins (E-cadherin and occludin).

*P. fusca* and *P. ficariae* were selected for this study based on several criteria. Firstly, their host plants are common worldwide, particularly in the study region. Secondly, both species (like other powdery and downy mildews) are known to produce an abundance of airborne spores, including conidia or sporangia as well as fragments of mycelium (hyphae) under favorable environmental conditions, which can be efficiently dispersed by wind [21]. Their frequent detection in air samples was confirmed by the authors in preliminary studies of fungal taxa from different systematic groups.

The natural consequence of these in vitro studies is investigating whether extracts from *P. fusca* and *P. ficariae* induce inflammatory and asthmatic reactions in mouse models of acute and chronic asthma. The research assessed inflammatory cytokines, specific IgE production, and alterations in lung tissue in affected mice.

## 2. Materials and Methods

### 2.1. Plant Material and Morphological Identification

The fungi selected for this research are biotrophic organisms that cannot be cultured on artificial media in laboratory conditions. Consequently, the specimens were collected directly from their natural habitats. Plant tissues infected by the microfungus from the Erysiphales (*Podosphaera fusca*) and the fungus-like species from the Peronosporales (*Peronospora ficariae*) orders were found in Puławy and Lublin, Poland. Specifically, *P. ficariae* was collected on *Ficaria verna* Huds. between 20–24 April and 21–27 April 2021, in Puławy and Lublin, respectively (LBL M-033124), while *P. fusca* was obtained from *Cucurbita pepo* in Ostrówek Podlaski near Łęczna on 29 September 2021, and 6 October 2021 (LBL M-033120). The host plant *Ficaria verna* Huds. typically grows in deciduous forests (especially riparian and oak-hornbeam forests), wet meadows, and thickets and is often found as a weed in parks and gardens. In contrast, *Cucurbita pepo* thrives in ruderal and segetal environments and is also widely cultivated as an agricultural crop. These plants are not under any species protection and are not located in protected areas.

**Figure 1:**
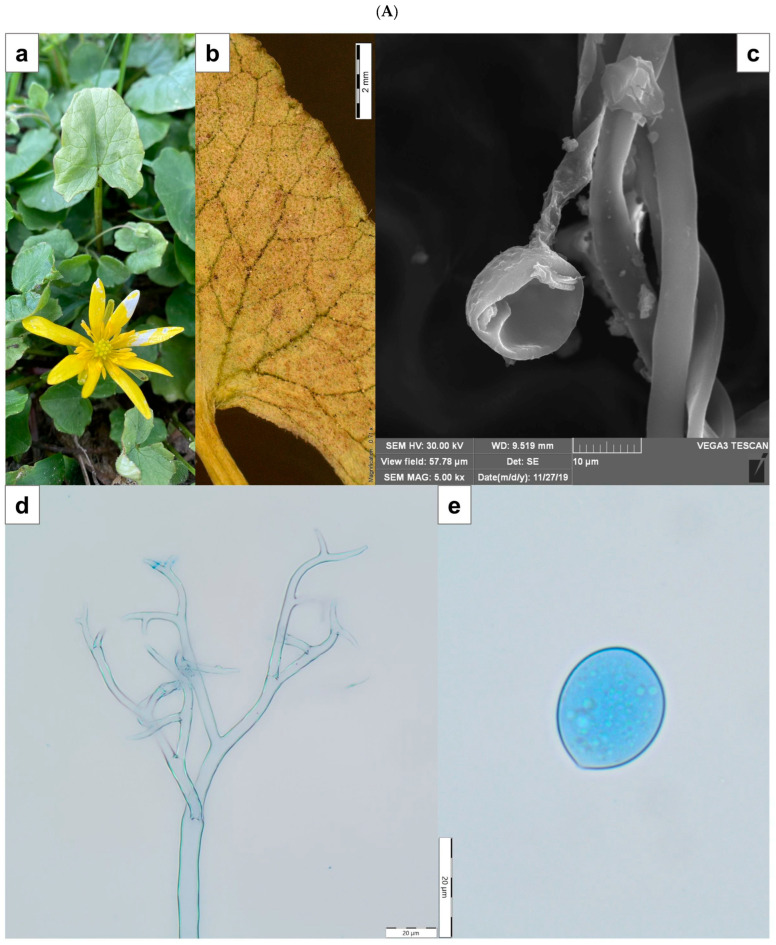

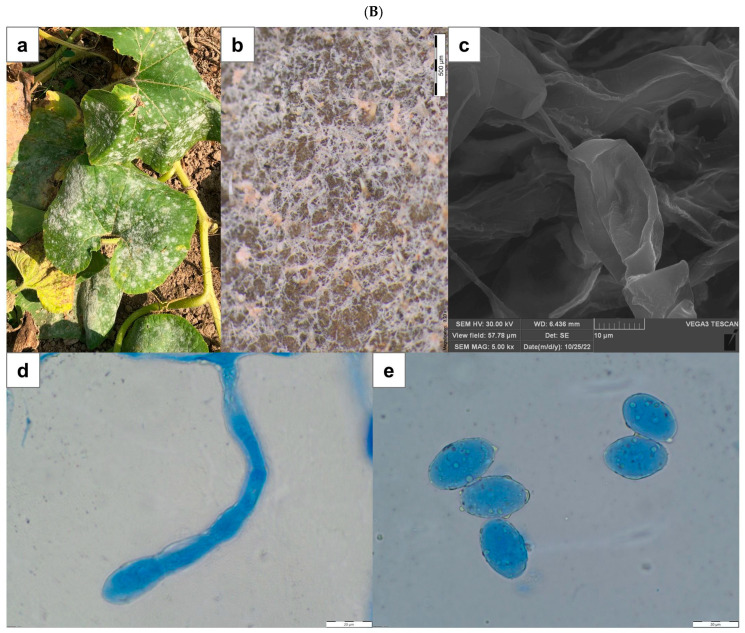
(**A**) *Peronospora ficariae* on *Ficaria verna.* (**a**)—disease symptom of chlorotic discoloration on the upper side of infected leaf; (**b**)—abundant sporulation on the lower leaf surface, composed of densely aggregated conidiophores (SM); (**c**)—produced conidium at the end of the conidiophore (SEM); (**d**)—apical branches of conidiophore (LM); (**e**)—conidium (LM). Scale: (**b**)—2 mm; (**c**)—10 µm; (**d**,**e**)—20 µm. (**B**) *Podosphaera fusca* on *Cucurbita pepo.* (**a**)—disease symptom on the upper side of infected leaves; (**b**)—coating composed of mycelium, conidiophores, and conidia visible on the leaf (SM); (**c**)—conidia (SEM); (**d**)—conidiophore with chain of conidia (LM); (**e**)—conidia (LM). Scale: (**b**)—500 µm; (**c**)—10 µm; (**d**,**e**)—20 µm. SM—stereoscopic microscope; LM—light microscope; SEM—scanning electron microscope.

The collected material, consisting of infected leaves and associated fungal structures, was air-dried and deposited in the herbarium at Maria Curie-Skłodowska University in Lublin. Initial morphological identification was conducted by mycologist U. Świderska, who prepared microscopic slides stained with Lactophenol Cotton Blue dye. Observations were conducted with an Olympus BX53 light microscope at magnifications of 40×, 100×, 400×, and 600×. Microphotographs of fungal diagnostic structures were captured using an Olympus digital camera SC180 attached to the microscope and an Olympus SZ10 stereoscopic microscope equipped with an Olympus camera XC50. Moreover, the structures were coated with gold using an Emitech K550X Sputter Coater (Quorum Emitech, East Grinstead, UK) and examined with a TESCAN Vega 3 LMU scanning electron microscope from Brno, Czech Republic (Figure 1A,B). Specimens exhibiting fungal morphological features, such as conidia and conidiophores, were then prepared for further laboratory analysis under the guidance of an Olympus SZ61 stereoscopic microscope. The samples were placed in test tubes and exposed to liquid nitrogen vapor for 24 h before grinding into a powder with a mortar and pestle. This powdered fungal material was used to produce crude extracts.

### 2.2. Preparation of Crude Fungal Extracts

The fungal samples underwent three acetone washes before dehydration at 37 °C for a full day. Once dried, the samples were suspended in 0.05 M Tris-HCl buffer (pH 8.0), using 1 mL of the buffer per 10 mg of dry weight. The suspension was subjected to three cycles of sonication in a room-temperature water bath (Elmasonic S100H, Elma, Singen, Germany), with each cycle consisting of 20 s of sonication followed by a 2 min cooling period. The extraction process was then continued overnight with stirring at 4 °C. Afterward, the extracts were centrifuged at 804× *g* for 10 min at 4 °C. The supernatants were transferred to a regenerated cellulose membrane with a 6–8 kDa molecular weight cutoff (Spectrum Laboratories, Rancho Dominguez, CA, USA) and dialyzed against 0.1 M ammonium bicarbonate (NH_4_HCO_3_) buffer (pH 8.4) for 24 h at 4 °C, with the dialysis solution replaced three times. Following the dialysis, the material was lyophilized and resuspended in phosphate-buffered saline (PBS, Biomed, Lublin, Poland). The protein concentration was determined using the Pierce BCA Protein Assay Kit (Thermo Fisher Scientific, Waltham, MA, USA), with bovine serum albumin (BSA) serving as a standard [22]. The resulting crude fungal extracts prepared in three independent replicates were divided into 100 µL aliquots and stored at −80 °C for future analysis.

### 2.3. Animal Model and Experimental Design

#### 2.3.1. Animals

The research involved adult female BALB/c/cmbd mice, aged 4–6 weeks, sourced from the Center for Experimental Medicine at the Medical University in Białystok, Poland. The animals were kept in the Animal House of the Department of Animal Physiology and Pharmacology, i.e., a certified facility within the Institute of Biological Sciences at Maria Curie-Skłodowska University in Lublin, Poland. Before the experiments began, the mice were given at least a week to acclimate to the new laboratory conditions. They were housed in groups of 8–9 in standard transparent cages equipped with environmental enrichment. The mice had unlimited access to regular rodent food and water. They were kept in controlled conditions, including a 12 h light/dark cycle starting at 6:00 a.m., a temperature range of 21–24 °C, relative humidity of 45–65%, and regulated air circulation. All procedures for handling and caring for the animals complied with Directive 2010/63/EU of the European Parliament and the Council of 22 September 2010 and with the Polish Act of 15 January 2015 on protecting animals used for scientific or educational purposes. The experimental protocol was approved by the Local Ethics Committee for Animal Experiments in Lublin, Poland (Approval No. 17/2023 of 30 January 2023).

#### 2.3.2. Study Design

Mice are frequently chosen as the subjects in acute and chronic asthma studies using animal models. In this research, female BALB/c/cmbd mice were divided into ten groups, each consisting of 8–9 mice. The experimental setup included five groups for acute asthma induction and another five for chronic asthma induction. As a positive control, a crude fungal extract was used alongside ovalbumin to induce both asthma types. The control groups received either an adjuvant (Imject^®^Alum, Thermo Fischer Scientific, Waltham, MA, USA) or PBS.

#### 2.3.3. Acute and Chronic Asthma Model

**Figure 2:**
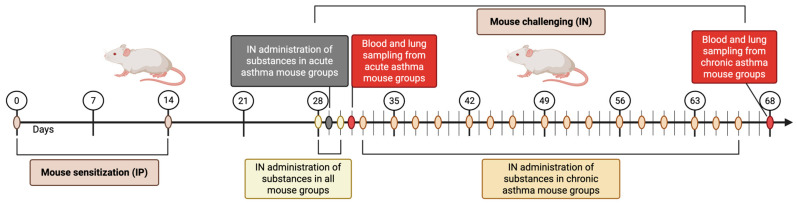
Chronological outline depicting the method for initiating and provoking both acute and chronic asthma in the experimental groups of mice (created with BioRender.com, accessed on 3 May 2025).

To create both acute and chronic asthma models, we adapted techniques from Kim et al. [23] and Daubeuf et al. [24]. The mice were sensitized intraperitoneally (IP) with ovalbumin (OVA), a control allergen, and with crude fungal extracts on days 0 and 14. Each mouse received 10 µg/mL of protein in 100 µL of PBS, which was adsorbed onto 100 µL of Al(OH)_3_ gel (Imject^®^Alum, Thermo Fischer Scientific, Waltham, MA, USA) (400 μg of protein/kg in a constant volume of 10 mL/kg) [24,25,26]. Subsequently, the mice were subjected to intranasal (IN) challenges with OVA and fungal crude extracts, receiving 50 µg of protein in 20 µL of PBS per mouse [25], as detailed in Table 1. Figure 2 illustrates the study timeline. For IN administration, the mice were placed in a small transparent container and anesthetized with 2.5% isoflurane using a rodent anesthesia system (SomnoSuite, Kent Scientific, Torrington, CT, USA). The anesthetized mice were held upright and administered 20 µL of PBS, OVA, or fungal extract into the nostril using a micropipette. To ensure that the liquid was distributed adequately in the airways, the mice were kept in an upright position for 30 s.

#### 2.3.4. Tissue Collection

Blood and lung samples were collected from the mice in both the acute and chronic asthma groups 24 h after the last exposure, specifically on days 31 and 68, as shown in Figure 1. The mice were euthanized by decapitation. Blood from the trunk was gathered in sterile Eppendorf tubes without any anticoagulant, and a single drop was used to create a blood smear on a glass slide. After clotting, the blood was stored at 4 °C for 24 h before centrifugation (314× *g*, 5 min, room temperature, RT). The sera were extracted and kept at −80 °C for later analysis. After decapitation, the lungs were extracted from the thoracic cavity, rinsed with ice-cold PBS, and immersed in 10% neutral buffered formalin (Sigma-Aldrich, St. Louis, MO, USA). Further experiments involving the biological materials (lungs and blood) were carried out in a BLS2-type laboratory of the Department of Virology and Immunology at Maria Curie-Skłodowska University in Lublin, Poland.

#### 2.3.5. Blood Smear Staining

After the drying stage, the smears were stained using the May-Grünwald-Giemsa method. Each smear was stained with 1 mL of a May–Grünwald stain solution (Sigma-Aldrich) for 3 min. Afterwards, 1 mL of distilled water was added to each slide, gently mixed with the stain, and left for another minute. The smears were then briefly rinsed with distilled water to remove any excess stain. Before proceeding to the next step, a 1:20 dilution of Giemsa stain (Sigma-Aldrich) was prepared using distilled water. Approximately 1.5 mL of the diluted stain was applied to each slide for 15 min. Subsequently, the smears were quickly rinsed again and left to dry. In the stained samples, leukocytes, including lymphocytes, granulocytes, eosinophils, and monocytes, were counted using an Olympus BX53 light microscope (Olympus, Münster, Germany) at a magnification of ×100. A hematological counter was used to count 200 cells, and the results were expressed as percentages of the leukocyte groups.

#### 2.3.6. Histological Analysis

The histological analysis of the lungs, focusing on eosinophil infiltration and airway remodeling, was performed at the Department of Clinical Pathomorphology, Medical University in Lublin. The left lung was preserved in formalin, embedded in paraffin, and sliced into sections. The sections, measuring 3–5 µm in thickness, were stained using hematoxylin-eosin (H&E) according to established protocols [27,28]. The staining procedure involved applying 500 μL of hematoxylin to the slide for 2 min at room temperature, followed by a 2 min rinse with gently flowing tap water. Next, 500 μL of eosin was applied for 1 min, followed by another rinse with tap water. The H&E-stained sections were utilized to assess the thickness of the airway epithelial and goblet cell layers and to investigate eosinophilic infiltration in both the control and treated mice after the allergen exposure. All histological samples were analyzed, photographed, and scored blindly. Images were captured using an Olympus BX40 microscope equipped with a U-TV0.63XC digital camera and Olympus cellSens Standard Software (version 1.13) (Olympus, Münster, Germany) at magnifications of ×4 or ×10.

### 2.4. Measurement of Cytokine Levels

Proinflammatory cytokine levels in mouse sera were quantified using ELISA (Invitrogen, Carlsbad, CA, USA), adhering to the manufacturer’s instructions. The measurements showed the levels of interleukin-4 (IL-4), interleukin-5 (IL-5), interleukin-13 (IL-13), TNF-α, and TGF-β proteins. The detection thresholds for these proteins were 2 pg/mL, 3.3 pg/mL, 2.8 pg/mL, 3.7 pg/mL, and 7.8 pg/mL, respectively.

### 2.5. Determination of Specific IgE Levels in Mouse Sera

The methods for detecting specific IgE antibodies employed in this study were established in our previous study [29]. Briefly, MaxiSorp immunoplates (Nunc, Roskilde, Denmark) were coated overnight at 4 °C with 100 µL per well of fungal crude extracts or ovalbumin at 1 µg/mL. After washing with PBS-T (0.05% Tween 20 in PBS, pH 7.4), the wells were blocked with 300 µL per well of PBS containing 3% BSA (Sigma, St. Louis, MO, USA) at 37 °C for 40 min. The supernatants were discarded, and the wells were washed three times with PBS-T. Subsequently, 100 µL of serially diluted serum samples were added to duplicate wells. The sera were diluted in PBS-T in a 1:2-fold series (1:64 to 1:65,536) for titration against the fungal antigen. BSA-coated wells served as blanks, while wells containing only fungal antigens were used to control nonspecific antibody binding. The plates were incubated at room temperature for 2 h and washed three times with PBS-T. To detect mouse IgE, 100 µL per well of appropriately diluted secondary anti-mouse antibodies conjugated to alkaline phosphatase were added. Goat anti-mouse IgE antibodies were sourced from Abcam (ab19967) (Cambridge, UK). After 2 h incubation at room temperature and three PBS-T washes, the alkaline phosphatase substrate (SigmaFast p-Nitrophenyl phosphate, 100 µL per well) was added and incubated for 30 min at room temperature. The reaction was halted with 1 M NaOH (100 µL per well), and absorbance at 405 nm was measured using a VICTOR X4 Multilabel Plate Reader (Perkin Elmer, Waltham, MA, USA). Specific IgE titers were determined as the reciprocal of the highest serum dilution factor that resulted in a mean absorbance value of 0.1.

### 2.6. Statistical Analyses

The Kolmogorov–Smirnov test was utilized to determine the data distribution characteristics. Based on the results, either parametric analyses (one-way ANOVA followed by the Tukey post hoc test) or non-parametric approaches (Kruskal–Wallis test with the Dunn multiple comparison test) were employed to evaluate statistical significance among the control and the studied factors for more than two samples. The Spearman rank correlation coefficient was used for correlation analysis. A *p*-value of less than 0.05 was considered statistically significant. Data analysis was conducted using STATISTICA software version 12 (StatSoft, Inc., Tulsa, OK, USA).

## 3. Results

### 3.1. Composition of Leukocytes in the Peripheral Blood of Mice

We employed one-way ANOVA to determine the F and *p* values for the mouse groups under study, including negative, positive (OVA), adjuvant control, *P. fusca*, and *P. ficariae* groups, in the acute and chronic asthma models. The analysis revealed statistically significant differences, as detailed in Table 2.

**Figure 3:**
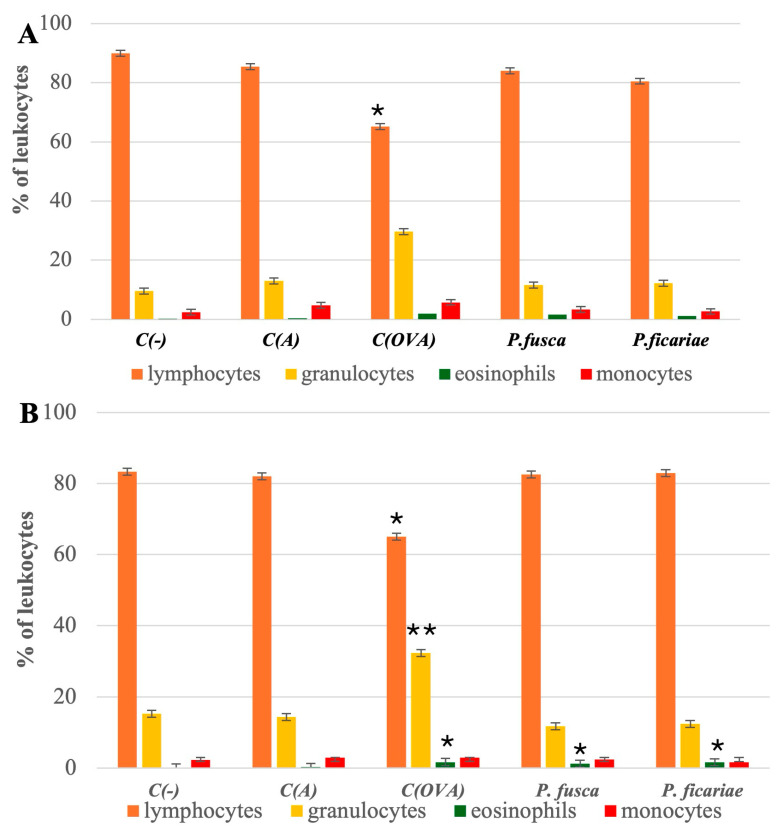
Percentage of individual groups of peripheral blood leukocytes in the mice studied. (**A**)—acute asthma model, (**B**) chronic asthma model. C(-)—negative control (PBS), C(A)—adjuvant control (Al(OH)_3_), C(OVA)—positive control ovalbumin (OVA). * statistically significant difference compared to the adjuvant control, * *p* ≤ 0.05, ** *p* ≤ 0.01; (one-way ANOVA with the Tukey post hoc test).

Compared to the percentage of lymphocytes in the blood of mice in the adjuvant control group (85.4 ± 1.9), the level of lymphocytes was significantly lower only after the induction of acute asthma with ovalbumin (62.25 ± 1.75). The percentage of lymphocytes after inhaling the *P. fusca* and *P. ficariae* extracts was comparable to that in the adjuvant control group. The group with the prolonged allergen exposure (chronic asthma model) exhibited similar results (Figure 3A,B).

In the acute and chronic asthma models, the blood granulocyte percentages only in the ovalbumin group (28.5 ± 1.6 and 32.3 ± 1.7, respectively) significantly exceeded the granulocyte levels in the blood of mice from the adjuvant group (13.0 ± 1.3 and 14.4 ± 2.1, respectively). After the intranasal inhalation of the *P. fusca* and *P. ficariae* extracts, the number of blood granulocytes was comparable to that in the adjuvant-treated mouse group in both acute and chronic asthma models (Figure 3A,B).

During acute asthma, all three allergens—OVA; *P. fusca*, and *P. ficariae*—caused a notable rise in blood eosinophil levels. However, with the extended exposure to the antigens, only OVA and *P. ficariae* significantly increased the count of these cells (Figure 3A,B). The proportion of monocytes remained consistent across the groups studied (Table 2). Overall, we found no differences in the blood leukocyte subpopulations between the acute and chronic asthma induction variants.

### 3.2. Cytokine Levels in Mouse Sera Following the Induction of Acute and Chronic Asthma

Using one-way ANOVA, we calculated the F and *p* values for all the mouse groups (negative, positive, and adjuvant controls and *P. fusca* and *P. ficariae* groups) in both the acute and chronic asthma models and found statistically significant differences, which are presented in Table 3.

**Figure 4:**
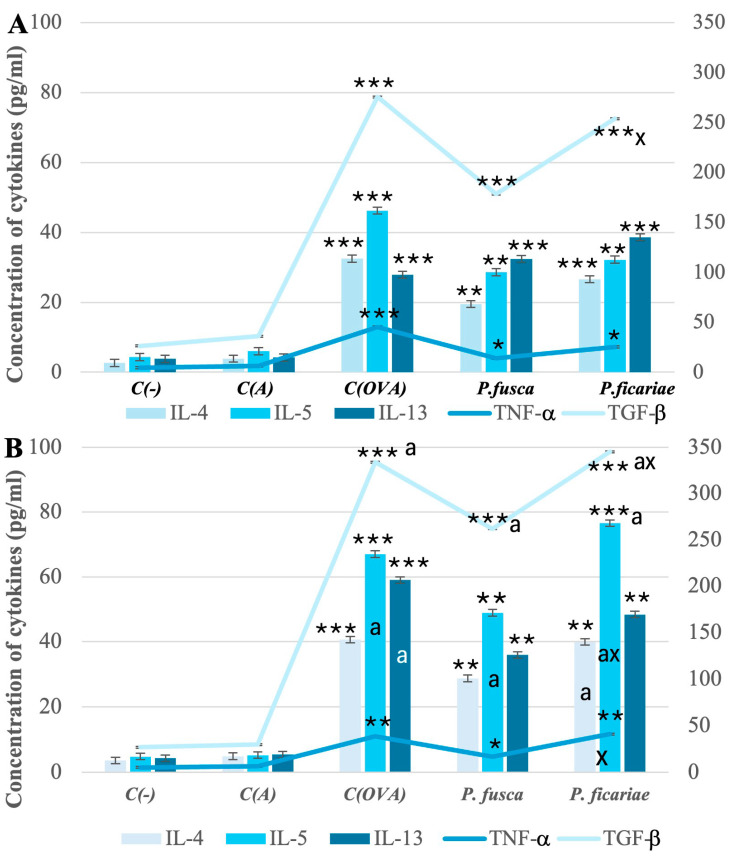
Levels of cytokines in mouse sera in acute (**A**) and chronic (**B**) asthma models. C(-)—negative control (PBS), C(A)—adjuvant control (Al(OH)_3_), C(OVA)—positive control ovalbumin (OVA). * statistically significant difference compared to the adjuvant control, * *p* ≤ 0.05, ** *p* ≤ 0.01, *** *p* ≤ 0.001; ^a^—statistically significant difference compared with the acute asthma model, ^a^ *p* ≤ 0.05, (one-way ANOVA with Tukey post hoc test), ^x^—statistically significant difference compared with the *P. fusca* variant, ^x^ *p* ≤ 0.05 (one-way ANOVA with Tukey post hoc test).

Mice with acute and chronic asthma exhibited markedly increased levels of IL-4, IL-5, IL-13, TNF-α, and TGF-β in their sera after exposure to ovalbumin and extracts from *P. fusca* and *P. ficariae.* However, after *P. ficariae* inhalation, the concentrations of TGF-β in the acute asthma model and TGF-β, IL-5, and TNF-α in the chronic asthma model were significantly higher than those in the *P. fusca* extract-exposed group (Figure 4A,B).

In the chronic asthma model, the extended exposure to such allergens as ovalbumin and fungal extracts led to significantly elevated levels of IL-5 and TGF-β compared to the acute asthma model. Furthermore, unlike *P. fusca, P. ficariae* triggered a greater increase in the IL-4 levels in the chronic than acute asthma model (Figure 4A,B).

However, it should be noted that, compared to the reference allergen OVA, the production of proinflammatory cytokines after using both fungal extracts was lower, although these differences were not statistically significant. One exception was IL-5 in the context of chronic asthma challenged by *P. ficariae*, where the cream titer slightly exceeded the level observed after using OVA.

### 3.3. Serum Levels of Allergen-Specific IgE in Mice After the Induction of Acute and Chronic Asthma

Using the Kruskal–Wallis test, we calculated the chi-square, df, and *p* values for all the mouse groups (negative, positive, and adjuvant controls and *P. fusca* and *P. ficariae* groups) in both the acute and chronic asthma models and found statistically significant differences, which are presented in Table 4.

**Figure 5:**
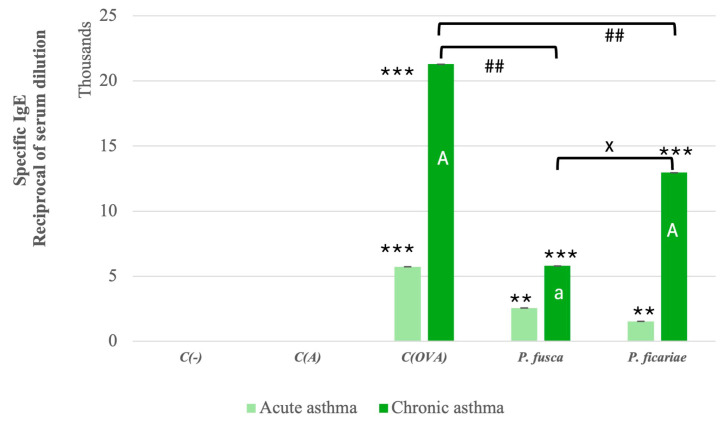
Levels of mouse sera IgE antibodies in acute and chronic asthma models. C(-)—negative control (PBS); C(A)—adjuvant control (Al(OH)_3_); C(OVA)—positive control ovalbumin (OVA). *—statistically significant difference compared to the adjuvant control, ** *p* ≤ 0.01, *** *p* ≤ 0.001; ^a^—statistically significant difference compared with the acute asthma model; ^A^ *p* ≤ 0.01, ^#^—statistically significant difference between ovalbumin and fungal extracts; ^##^ *p* ≤ 0.01 (Kruskal–Wallis test followed by the Dunn multiple comparison test); ^x^—statistically significant difference compared with the *P. fusca* variant; ^x^ *p* ≤ 0.05 (one-way ANOVA with Tukey post hoc test).

In both acute and chronic asthma models, we observed a statistically significant increase in the production of specific IgE antibodies recognizing ovalbumin and proteins from the *P. fusca* and *P. ficariae* extracts. Notably, a more substantial level of IgE production was detected following the prolonged exposure of the animals to the three allergens. However, the extracts from *P. fusca* and *P. ficariae* were significantly less effective than ovalbumin in inducing specific IgE, with *P. fusca* being much less effective than *P. ficariae* in promoting IgE production during extended contact (Figure 5). Considering the role of IL-4 in IgE production, we assessed the Spearman correlation between the IgE and IL-4 levels. Our results showed a significantly positive correlation during chronic asthma development for the OVA (r = 0.88, *p* ≤ 0.05), *P. fusca* (r = 0.925, *p* ≤ 0.05), and *P. ficariae* (r = 0.878, *p* ≤ 0.05) allergens.

### 3.4. Histological Analyses of Mouse Lungs

**Figure 6:**
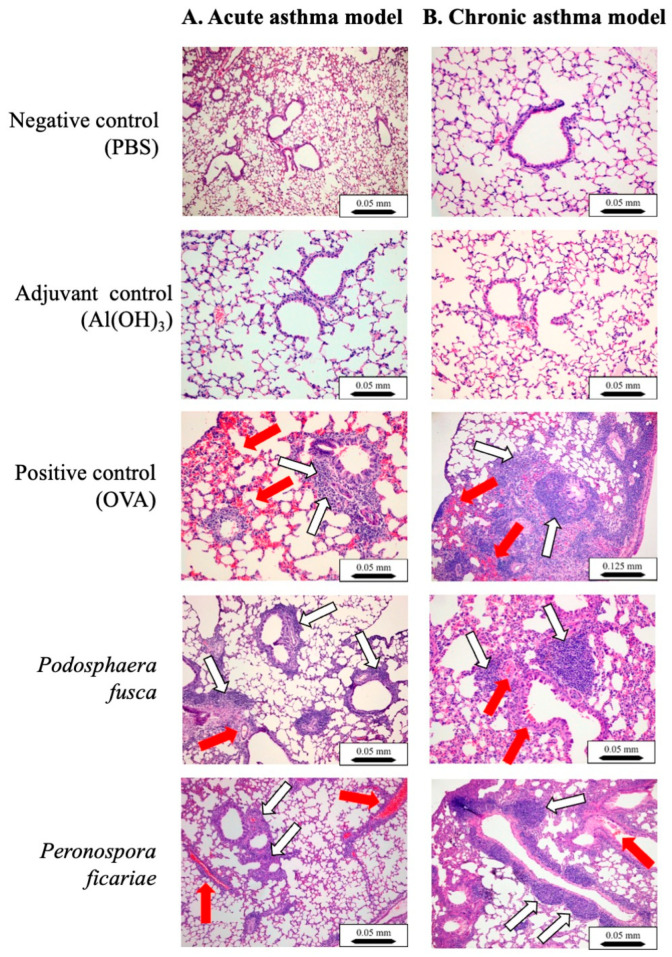
Anatomical and structural mouse lung lesions following sensitization and asthma challenges. Paraffin-embedded lung sections were stained with H&E and examined using a light microscope. The lung tissue showed localized recruitment of eosinophils (indicated by red arrows) as well as lymphocytes, macrophages, and neutrophils (indicated by white arrows). Scale bar: 0.05 mm (×10 magnification) or 0.125 mm (×4 magnification).

In both the negative and adjuvant control groups, the lung microscopic structure remained unchanged, regardless of whether the asthma was acute or chronic (Figure 6, panels A and B). In the acute asthma model’s positive control (OVA), dense inflammatory infiltrates were noted in multiple peribronchiolar, peribronchial, and perivascular regions. These infiltrates were primarily composed of neutrophils and eosinophils, accompanied by lymphocytes and macrophages. Five animals exhibited focal interstitial pneumonia, predominantly in the central (parabronchial) lung area. The bronchial and bronchiolar epithelial layers showed mild hyperplasia. Furthermore, congestion and focal interalveolar hemorrhages were observed (Figure 6, panel A).

In the chronic asthma model, the positive control (OVA) exhibited similar multifocal inflammatory infiltrates, although the composition was slightly different, with a predominance of plasma cells and lymphocytes. Eosinophils and neutrophils were still present. Focal pneumonia was more widespread, affecting seven animals, and was located in the same area as the acute group. There were focal interalveolar accumulations of foamy macrophages. Multifocal subpleural accumulations of small lymphocytes were found; they were particularly abundant in two mice. Severe thickening of small blood vessel walls was observed in eight animals. Mild hyperplasia of the bronchiolar epithelial layer was noted. Congestion and focal interalveolar hemorrhages were also observed (Figure 6, panel B).

Following the sensitization and intranasal exposure to the *P. fusca* extracts in the acute asthma model group, the lung damage was similar to that seen in the positive control (OVA) in the acute asthma model. However, it was less pronounced (Figure 6, panel A). Two mice in this group showed more widespread pneumonia. In the chronic asthma group, the lung damage mirrored that of the positive control group in the chronic asthma model but was less severe. One mouse exhibited extensive pneumonia. In both the acute and chronic asthma models, there was a buildup of eosinophils, lymphocytes, and granulocytes in the lungs (Figure 6, panels A and B).

In the acute asthma model, the mice were sensitized and challenged intranasally with the *P. ficariae* extract, resulting in reactions similar to those caused by ovalbumin and *P. fusca*, such as significant leukocyte infiltration (Figure 6, panel A). Two mice from the *P. ficariae* group developed more severe pneumonia. The examination of the lung tissue from these mice showed lesions that were identical in nature and severity to those found in the positive control (OVA) group (Figure 6, panel B).

## 4. Discussion

In developed countries, allergic conditions are regarded as the epidemics of the 21st century, impacting over 30% of the population with a swiftly increasing prevalence. Among the various sources of allergens, fungi are particularly significant. There are 174 fungal allergens identified within the Ascomycota phylum and 30 within the Basidiomycota phylum. The main allergenic fungi belong to the genera *Alternaria*, *Aspergillus*, *Cladosporium*, *Penicillium*, and *Fusarium* [30,31]. However, patients are not always specifically diagnosed with the allergens they react to. For the first time, we introduce two plant parasites, *Podosphaera fusca*, a microfungus from the Erysiphales order, and *Peronospora ficariae*, a fungus-like organism from the Peronosporales order, which exhibit allergenic potential in murine models of acute and chronic asthma. These findings strongly support our previous research using in vitro models of the upper (BEAS-2B cell line) and lower (A549 cell line) respiratory tracts, where *P. fusca* and *P. ficariae* demonstrated pro-inflammatory and pro-allergenic potential (unpublished data).

The Global Asthma Report indicates that asthma affects 9.1% of children, 11.0% of adolescents, and 6.6% of adults worldwide. This respiratory ailment has a profound impact on patients and their families globally, presenting significant psychological, medical, and financial difficulties for those involved [32]. Asthma is primarily caused by an inflammatory reaction in the respiratory system to usually harmless environmental substances, both inorganic and organic, as well as by genetic factors. The chronic inflammation and clinical symptoms linked to asthma occur when interactions between inflammatory and resident cells initiate a series of events [1,5,33]. Considering the crucial role of white blood cells in immune and inflammatory processes, we examined the variations in specific leukocyte populations in the blood and lungs of mice with acute or chronic asthma compared to healthy control subjects.

Studies have consistently demonstrated that high levels of lymphocytes and eosinophils are uniformly present in the airways of individuals with asthma, irrespective of the severity of the condition. Moreover, the presence of eosinophils might be linked to the intensity of asthma symptoms [10,34,35,36]. In contrast, our research utilized both acute and chronic asthma models and identified a notable reduction in blood lymphocytes only after the administration of ovalbumin. This observation implies that these white blood cells migrate to the respiratory tract and are influenced by the method of allergen exposure. Our analysis of lung samples from allergic mice showed a significant influx of lymphocytes, particularly following the exposure to ovalbumin. Nonetheless, both *P. fusca* and *P. ficariae* also induced a minor increase in the lymphocyte count.

Although eosinophilic inflammation is a hallmark of asthma progression, neutrophilic inflammation is also significant. In certain instances, co-occurrence of both eosinophils and neutrophils can be found. Based on the relative abundance of these cell types in sputum samples, asthma is categorized into three types: eosinophilic asthma, neutrophilic asthma, and mixed neutrophilic-eosinophilic asthma [8,37].

Our research found that the blood neutrophil levels increased solely in response to the classical asthma trigger, ovalbumin, in both acute and chronic disease models. Following the allergization with *P. fusca* and *P. ficariae*, the blood neutrophil percentages were similar to those in the adjuvant control group. Nonetheless, it is possible that blood neutrophils migrated to the lungs, as confirmed by our histological examination in both acute and chronic asthma models. Unlike neutrophils, a significant infiltration of eosinophils in the blood and lungs was noted after the sensitization with both fungi, although it was less intense than in the positive control (OVA).

Numerous fungal species facilitate the movement of eosinophils, lymphocytes, and neutrophils into the lungs. In a study by Lilly et al., it was observed that mice subjected to repeated exposure to *A. fumigatus* in an experimental model of fungal asthma exhibited a predominance of eosinophils in their lungs. The research also highlighted that, during acute challenges with *A. fumigatus*, neutrophils became the dominant cell type in the lungs [34]. Janssens et al. confirmed the influx of eosinophils and neutrophils into the lungs in a murine model after exposure to *A. fumigatus* as well [38]. In another mouse model of allergy triggered by *A. alternata* and *Cladosporium herbarum*, scientists noted pulmonary inflammation marked by increased lung neutrophils, eosinophils, and lymphocytes [39].

Asthma is a persistent inflammatory condition of the airways linked to type 2 cytokines, such as IL-4, IL-5, and IL-13. These cytokines contribute to several asthma characteristics: increased eosinophil presence in the airways, heightened mucus production, greater bronchial sensitivity, and the generation of IgE antibodies, another sign of allergic inflammation [40]. In asthma patients, eosinophils and CD4+ lymphocytes that secrete IL-5 are often detected in blood samples and fluid from lung lavage procedures [41,42,43]. *A. fumigatus* is the most common fungal organism causing allergic bronchopulmonary mycosis and asthma. Research has shown that *A. fumigatus* triggers a Th2 response, particularly producing IL-4, IL-5, and IL-13. IL-4 from Th2 cells affects B cells, promoting the switch from IgG to IgE. IL-5, which is essential for eosinophilic inflammation, aids in the recruitment, growth, and maturation of eosinophils (and basophils). At the same time, IL-13 induces airway hyperresponsiveness (AHR), stimulates goblet cells, and increases mucus secretion [40,41,44]. Both IL-4 and IL-13 inhibit the expression of E-cadherin and occludin, which form tight junctions between respiratory epithelial cells [45].

In experiments involving mice, the administration of OVA, a model antigen, combined with aluminum hydroxide (a compound that encourages Th2 cell development) into the peritoneal cavity led to the formation of OVA-specific Th2 cells. These cells secreted IL-4, IL-5, IL-10, and IL-13, along with antigen-specific IgE [41]. We also employed the model OVA antigen to compare the effects of *P. fusca* and *P. ficariae*. Both fungal extracts significantly elevated the IL-4, IL-5, IL-13, TNF-α, and TGF-β levels in mice with acute and chronic asthma. In comparison to the acute asthma model, the prolonged exposure to all the allergens (OVA, *P. fusca*, and *P. ficariae*) resulted in a notable increase in IL-5 and TGF-β levels, with a more pronounced response observed following the *P. ficariae* exposure than the *P. fusca* inhalation in both asthma models. Furthermore, we noted a significant increase in the serum IL-4 concentration during the induction of chronic asthma reactions in mice. However, regarding the induction of a pro-inflammatory response, OVA was the most efficient allergen compared to both fungal extracts.

Studies have shown that TNF-α, which is found in higher levels in severe asthma cases, contributes to the disruption of barrier function and the activation of cells within bronchial epithelial tissue [46]. Increased levels of this cytokine are associated with a rise in reactive oxygen species (ROS) production, which weakens the bonds between bronchial epithelial cells by decreasing the expression of E-cadherin and occludin. Our laboratory experiments clearly demonstrated that *P. fusca* and *P. ficariae* significantly enhanced TNF-α and ROS production in models of both the upper (BEAS-2B cell line) and lower (A549 cell line) respiratory tract (unpublished data). This increase was positively associated with the breakdown of epithelial cell junctions, as indicated by the presence of E-cadherin and occludin. During the acute phase of asthma, the levels of TNF-α induced by inhaling *P. fusca* and *P. ficariae* were lower than those in the ovalbumin group. However, in the chronic asthma model, the serum concentration of this cytokine was similar among the animal groups exposed to OVA and *P. ficariae* allergens.

Studies have indicated that transforming growth factor beta (TGF-β) and Th2 cytokines (IL-5 and IL-13) contribute to airway remodeling as asthma progresses [42,45]. Numerous studies have reported increased TGF-β activity in asthmatic conditions [47,48]. TGF-β affects airway remodeling through various mechanisms, such as promoting the proliferation of airway smooth muscle (ASM) cells and inducing epithelial cell apoptosis. Additionally, TGF-β has strong profibrotic effects on mesenchymal cells. Research using an animal model of allergic asthma has shown that this growth factor boosts extracellular matrix deposition, encourages ASM cell growth, and enhances mucus production [49,50]. Namvar et al. found that *A. fumigatus* conidia (strain Af293) triggered the production of key profibrogenic growth factors, specifically TGF-β1 and TGF-β2, in primary airway epithelial cells [51]. Furthermore, mice exposed to *A. fumigatus* spores showed elevated TGF-β levels linked to significant inflammation and airway remodeling [52]. Our studies observed notably high TGF-β concentrations following the intranasal exposure to the three allergens studied, although the *P. fusca* extract was less effective than OVA and *P. ficariae*. Moreover, compared to acute asthma, the levels of this cytokine were significantly higher during the chronic phase of the disease.

The role of IgE antibodies in the onset of allergic conditions, such as asthma, is well-established. In the typical immediate hypersensitivity reaction, allergens with multiple binding sites connect IgE antibodies attached to mast cells via the high-affinity IgE receptor (FcϵRI). This interaction results in the release of pre-stored vasoactive substances, triggers cytokine transcription, and encourages the formation of new prostaglandins and leukotrienes. Within the respiratory system, these chemical mediators swiftly cause swelling in the bronchial mucosa, enhance mucus production, and lead to smooth muscle contraction. Consequently, they attract inflammatory cells to the affected area [53,54]. Fungal elements can act as allergens, initiating an IgE-mediated immune response that plays a role in the onset and exacerbation of asthma [31,55]. For example, increased levels of specific IgE were found in asthma patients where *A. fumigatus* was identified as a contributing factor [56,57].

Furthermore, research has shown a strong correlation between IgE levels and IL-4 production following *A. fumigatus* exposure in both mice [44,58] and humans [59]. The extracts from *P. fusca* and *P. ficariae* significantly stimulated specific IgE production in the mouse models of both acute and chronic asthma. However, the IgE levels were considerably lower than those observed after the intranasal ovalbumin exposure, and the IgE levels after the *P. ficariae* challenge were significantly higher than in the *P. fusca* variant. Additionally, we observed a strong positive correlation between the IL-4 production and the blood IgE levels following the exposure to the three allergens in the chronic asthma model. Although detecting IgE antibodies in mouse serum directed against fungal extracts indicates the specificity of the reaction, it remains unclear which exact components of the extract the IgE was directed toward—the proteins themselves or their combinations with lipids—this requires further studies.

The airway epithelium acts as a complex barrier, providing physical, functional, and immune protection to the body from potentially harmful airborne particles. This defense is essential for maintaining the host’s health. However, when this protective barrier is compromised, immune and inflammatory responses to external allergens, microbes, and pollutants can be triggered. This susceptibility may increase the risk of developing chronic inflammatory conditions like allergic rhinitis, chronic rhinosinusitis, and asthma. In these upper airway diseases and asthma, the airway epithelium becomes dysfunctional due to the impaired formation of tight junctions through E-cadherin and occludin [35,51,52,60,61]. Indeed, during the progression of allergic asthma, the airway undergoes remodeling that starts with epithelial changes, such as epithelial shedding, destruction of ciliated cells, and epithelial cell hyperplasia [62].

Airway remodeling plays a significant role in the clinical manifestations of chronic asthma. This process involves structural changes in large and small airways, impacting cellular elements and the extracellular matrix. The remodeling includes the death of epithelial cells, increased proliferation of airway smooth muscle cells, and activation of fibroblasts. Complex interactions among various cell types, such as eosinophils, neutrophils, and lymphocytes, drive these structural transformations in the airway wall and submucosa. The pathological changes that result from this process contribute to the progression and symptoms of chronic asthma [62,63,64].

Labram et al. found that, when exposed to *A. fumigatus* spores, human and mouse airway epithelial cells generated pro-fibrogenic factors like TGF-β1, TGF-β2, periostin, and endothelin-1. These factors led to significant inflammation and remodeling of the airways, as evidenced by the loss of epithelial cells, subepithelial fibrosis with increased extracellular matrix buildup, pronounced smooth muscle enlargement, and an increase in goblet cell numbers [52].

Our research concentrated on examining inflammatory symptoms in the lungs of mice following the inhalation of fungal extracts from *P. fusca* and *P. ficariae.* In the histological samples, we observed alterations akin to those caused by ovalbumin, although they were less pronounced in both acute and chronic asthma models. We also detected dense inflammatory infiltrates composed of neutrophils and eosinophils in multifocal areas around the bronchioles, bronchi, and blood vessels, along with mild hyperplasia of the bronchial and bronchiolar epithelial layers and significant thickening of the walls of small blood vessels. While focal pneumonia was widespread in the OVA-positive group, isolated cases of pneumonia were also noted in the groups treated with *P. fusca* and *P. ficariae.*

## 5. Conclusions

This research presents two previously unexamined plant pathogens: the fungus-like organism *Podosphaera fusca* and the microfungus *Peronospora ficariae.* These plant parasites triggered an inflammatory response in a mouse model of both acute and chronic asthma; however, that response was weaker than the effects of the well-known allergen ovalbumin. The results revealed the production of inflammatory cytokines, specific IgE antibodies, and noticeable inflammatory changes in the murine lungs following intranasal exposure to the fungal allergens, allowing us to speculate about the potential proasthmatic nature of the tested fungal extracts. Our study broadens the range of known fungal allergens, which could be vital for diagnosing respiratory allergies. Additionally, the host plants of these parasites, such as the medicinal perennial *Ficaria verna* and the cultivated plant *Cucurbita pepo*, are frequently found in human environments. Further investigation is needed to determine whether individuals with previously undiagnosed asthma have allergic reactions to the fungi studied here.

## 6. Study Limitations

Our study is limited because it uses basic microfungal extracts, which need standardization to identify the most active components, such as proteins, fatty acids, or their complexes. Another essential methodological limitation is the absence of a control extract from uninfected host plants. 

## Figures and Tables

**Table 1:** Method for inducing and initiating both acute and chronic asthma in the studied mouse groups.

Group	Sensitization	Challenge	
	Day 0 and 14 (IP)	Acute Asthma	Chronic Asthma
		Days 28, 29, and 30 (IN Under General Anesthesia)	3× per Week for 6 Weeks Starting from Day 28 (IN Under General Anesthesia)
Negative control (PBS)	PBS	20 µL PBS/mouse	20 µL PBS/mouse
2. Adjuvant control (Al(OH)_3_)	PBS/Al(OH)_3_ (1:1)	20 µL PBS/mouse	20 µL PBS/mouse
3. Positive control (OVA)	OVA (400 μg/kg) in PBS/Al(OH)_3_ (1:1)	OVA (conc. 1 mg of protein/mL PBS)—20 μL/mouse	OVA (conc. 1 mg of protein/mL PBS)—20 μL/mouse
4. *Podosphaera fusca*	Fungal extract (400 μg of protein/kg) in PBS/Al(OH)_3_ (1:1)	Fungal extract (conc. 1 mg of protein/mL PBS)—20 μL/mouse	Fungal extract (conc. 1 mg of protein/mL PBS)—20 μL/mouse
5. *Peronospora ficariae*			

**Table 2:** F and *p* values for blood leukocytes compared among all the mouse groups (one-way ANOVA).

Leukocyte Subpopulations	Acute Asthma Model		Chronic Asthma Model	
	F	*p*	F	*p*
Lymphocytes	146 (3, 42)	0.001	83.4 (5, 33)	0.001
Granulocytes	216 (15, 38)	0.001	246.8 (10, 45)	0.001
Eosinophils	4.66 (1, 3)	0.004	6.16 (1, 5)	0.04
Monocytes	1.06 (1, 3)	0.25	1.94 (1, 2)	0.12

**Table 3:** F and *p* values for serum cytokine levels compared among all the mouse groups (one-way ANOVA).

Cytokine	Acute Asthma Model		Chronic Asthma Model	
	F	*p*	F	*p*
IL-4	296.8 (20, 62)	0.001	280.3 (35, 65)	0.002
IL-5	441 (29, 47)	0.001	1 508 (48, 86)	0.004
IL-13	364.5 (32, 56)	0.002	522 (29, 66)	0.001
TNF-α	452.6 (31, 54)	0.007	420 (25, 52)	0.005
TGF-β	1 130 (33, 51)	0.001	1 241 (36, 75)	0.005

**Table 4:** Chi-square, df, and *p* values for serum IgE levels compared among all the mouse groups (Kruskal–Wallis test).

Ig	Acute Asthma Model			Chronic Asthma Model		
	Chi-Square	df	*p*	Chi-Square	df	*p*
IgE	17.0	6	0.009	18.75	6	0.008

## Data Availability

The original data are available in the Zenodo repository under the DOI: 10.5281/zenodo.15655974.

## Abbreviations

The following abbreviations are used in this manuscript:

NCD	Noncommunicable Disease
ELISA	Enzyme-Linked ImmunoSorbent Assay
OVA	OVAlbumin
ASM	Airway Smooth Muscle
